# External quality assessment of malaria microscopy diagnosis among public health facilities in West Amhara Region, Ethiopia

**DOI:** 10.1186/s13104-017-3080-0

**Published:** 2017-12-21

**Authors:** Hiwot Amare Hailu, Melashu Balew Shiferaw, Leykun Demeke, Mulatu Melese Derebe, Zelalem Dessie Gelaw, Manamnot Agegne Emiru, Mastewal Worku Lake

**Affiliations:** 1 Ethiopian Public Health Institute, Addis Ababa, Ethiopia; 2 Amhara Public Health Institute, Bahirdar, Ethiopia; 3 International Center for AIDS Care and Treatment Programs, Columbia University, Addis Ababa, Ethiopia

**Keywords:** Malaria, External quality assessment, Amhara Public Health Research Institute

## Abstract

**Objective:**

To evaluate the importance of external quality assessment program on malaria microscopic diagnosis.

**Results:**

A total of 3148 slides were collected in 4 consecutive external quality assessment rounds and blindly rechecked at Amhara Public Health Institute. The average agreement between health facility and APHI slide readers was 96.6%. The percent agreement for parasite detection and species identification for *P. falciparum* became improved in four consecutive EQA rounds from 93.88 to 99.24% and 92.67 to 97.35% respectively. The rates of false positive and false negative were also dramatically decreased in each round from 10.5 to 0.79% and 2.14 to 0.74% respectively. Therefore, we recommend that malaria EQA program should maintain and expand in all malaria diagnostic health facilities in the region to provide accurate and reliable malaria microscopic service.

## Introduction

Malaria is a serious public health problem in many parts of the world, demanding an unacceptable toll on the health and economic welfare of the world’s poorest communities [1]. Annual malaria cases in Ethiopia are estimated to be around 5 million cases per year. Its transmission is unstable and seasonal [2].

Prompt and accurate diagnosis of malaria is part of effective disease management that is based on clinical suspicion and detection of parasites in the blood [3]. Blood smear microscopy diagnosis is the most affordable, accessible and reliable technique for diagnosis of malaria infection [4].

Failure to detect persons with malaria can lead to the continued spread of infection in the community, miss use of anti-malaria drugs and fasten the appearance of drug resistance [3]. Active quality assurance program, competent and motivated staff, effective training, regular mentoring and adequate supply will ensure uninterrupted and quality malaria microscopic diagnosis service [5].

External quality assessment (EQA) is a quality assurance method for objectively checking the laboratory’s performance through panel testing, blinded rechecking of slides and on-site supervision. The blind rechecking method is a process by which slides are randomly selected from diagnostic health centers, transported to the higher level laboratory and reexamined blindly to evaluate the performance of laboratories [6]. Studies done by Pakistan and Congo showed that external quality assessment methods were found to be feasible and acceptable to improve the quality of malaria microscopy service [7, 8]. The major deficiencies may be due to poor competency, poor equipment, poor reagents, or poor infrastructure and work practices that require urgent alteration [7]. EQA program ensures reliable malaria microscopic diagnosis that recognized as an important component of effective malaria case management and control. However, an EQA program has not been conducted previously and not recognized as an important method to improve the quality of malarial microscopic diagnosis services in the study area. Therefore, this study had an objective of evaluating the importance of external quality assessment program on the improvement of malaria microscopic diagnosis at health center laboratories in the region.

### Main text

#### Methods

An institutional based, cross-sectional study was conducted from July 2013 to October 2014. The study was carried out at malaria diagnostic health centers in West Amhara region. The region has five zones and two city administrations that have a total population of 11,240,275 [9]. There were 8 hospitals, 278 diagnostic health centers and one research institute in the region which provide different health care services. EQA program of malaria has been coordinated by Amhara Public Health Institute (APHI).

A total of 20 health centers were enrolled and participated in this program and all were included in this evaluation. In accordance with the national malaria EQA guideline, 40 giemsa stained slides (20 positive and 20 negative) were randomly selected from each participated diagnostic sites in every 4 months. A total of 4 consecutive EQA rounds conducted from July 2013 to October 2014. All the selected slides with their result from each site sent to APHI for rechecking in every round. Then slides were distributed to trained quality officers at APHI without disclosing the result of health centers. When discrepant results identified, the slides were rechecked by senior quality officer for confirmation and the result was considered as final. Discordant slides then taken to their corresponding diagnostic sites by APHI experts to show their errors and trained practically on site to improve the skill of the lab staff of diagnostic sites. Additionally, APHI has prepared and sent a feedback in the form of a written report; showing details of gaps and offering suggestions to improve the quality of diagnosis services after each round of rechecking. The discordant result stands for any positive result reported as negative, or any negative result reported as positive, or any species misdiagnosis.

Data were entered and analyzed using Microsoft Office Excel 2007. Each laboratory was evaluated for percentage of agreement, false positive rate, false negative rate and species misdiagnosis. The trend of the performance of each laboratory also calculated. Finally, the result was presented with tables, graphs, and figures.

Ethical clearance was secured from the Amhara Regional Health Bureau Ethical Review Committee. Official permission was also obtained from each participated health centers. The result was kept confidentially and communicated to Amhara Regional Health Bureau, Amhara Public Health Institute, Ethiopian Public Health Institute and other partners.

### Result

#### Agreement of results

A total of 3200 slides were collected in 4 consecutive EQA rounds in 16 months period from 20 health facility laboratories. Of which 52 slides were damaged during transportation and in the process. Out of 3148 slides, 1487 slides were reported as positive and 1661 slides were reported as negative at participated health facilities laboratories. All collected slides were rechecked at APHI and 1402 slides reported as positive and 1639 slides were reported as negative. Out of the total collected slides, the result of 96.6% was agreed between the participated health facilities and APHI readers. However, the result of 3.4% slides was discordant. As the EQA program has continued from round one to four the number of discordant slides was decreased notably (see Table 1).

**Table 1:** Overall agreement between participated health facilities and APHI slide readers in consecutive EQA rounds from July 2013 to October 2014

EQA rounds	Total slides	1st Reader (health facility)		2nd Reader (APHI)		3rd Reader (APHI)		Agreed result	Discrepant result	% Agreement
		Positive	Negative	Positive	Negative	Positive	Negative			
Round 1	801	385	416	345	407	345	407	752	49	93.88
Round 2	797	364	433	335	425	335	425	762	35	95.36
Round 3	762	357	405	344	403	344	403	746	16	97.77
Round 4	788	381	407	378	404	378	404	782	6	99.24
	3148	1487	1661	1402	1639	1402	1639	3042	106	96.62

The percent agreement result between participated health facilities and APHI slide readers improved in each 4 consecutive EQA rounds (94, 95, 98 and 99%). Species identification percent agreement for *P. falciparum,* between participated health facilities and APHI readers, was also improved, from 92 to 97%, as the implementation of EQA program has been continued.

#### False negative, false positive and species misdiagnosis

There were a total of 213 discordant slides of which 107 slides were species misdiagnosed, 83 slides were false positive and 23 slides were a false negative. The number of discordant slides in all disagreement types was decreased with continued EQA implementation (see Table 2).

**Table 2:** Total discordant results between participated health facilities and APHI slide readers in consecutive EQA rounds from July 2013 to October 2014

EQA rounds	Discordant types			Total discordant
	False positive	False negative	Species misdiagnosis	
Round 1	40	9	29	78
Round 2	27	8	40	75
Round 3	13	3	23	39
Round 4	3	3	15	21
Total	83	23	107	213

A total of 546 *P. falciparum* blood film slides were reported as *P. falciparum* whereas 36 *P*. *falciparum* blood film slides were read as *P. vivax*, mixed and negative. Seven hundred forty-eight (748) *P. vivax* blood film slides were reported as *P. vivax* correctly whereas 90 *P. vivax* blood film slides were read as *P. falciparum*, mixed and negative. A total of 1644 negative blood films were read as negative whereas 83 negative giemsa stained blood films were read as *P. falciparum*, *P. vivax* and mixed. Eight-five giemsa stained blood film slides were misdiagnosed as *P. vivax* which was the most frequent followed by 64 blood film slides were misdiagnosed as *P. falciparum* (see Table 3). The false positive rate was dramatically decreased in each malaria EQA rounds from 10.5 to 0.79%.

**Table 3:** Result of species identification between participated health facility and APHI slide readers in West Amhara Region from July 2013 to October 2014

Slide reading results	Four consecutive EQA blind rechecking rounds				Total
	Round 1	Round 2	Round 3	Round 4	
*P. f* as *P. f*	153	121	93	179	546
*P. f* as *P. v*	5	2	7	3	17
*P. f* as mixed	4	3	2	4	13
*P. f* as negative	2	2	0	2	6
*P. v* as *P. v*	155	174	244	175	748
*P. v* as *P. f*	7	26	10	3	46
*P. v* as mixed	12	9	2	4	27
*P. v* as negative	7	6	3	1	17
Mixed as mixed	4	0	4	9	17
Mixed as *P. f*	0	0	1	1	2
Mixed as *P. v*	1	0	1	0	2
Mixed as negative	0	0	0	0	0
Negative as negative	411	427	402	404	1644
Negative as *P. f*	8	3	4	1	16
Negative as *P. v*	31	24	9	2	66
Negative as mixed	1	0	0	0	1
Total	801	797	762	788	3148

P. f: *Plasmodium falciparum*, P. v: *Plasmodium vivax*, Mixed: *P. f* and *P. v*

#### Discussion

Immediate and long-term clinical, public health and health planning decisions for malaria control are based on laboratory test results. Incorrect delayed or misinterpreted tests can have serious consequences for patients and communities; undermine confidence in the service and waste scarce resources. Rechecking may detect malaria misdiagnosis in routine work and assess the overall quality of testing. This should not be considered a criticism of the person who performed the routine examination [5].

Prior to the implementation of external quality assessment program in the participated health facilities, training was provided on malaria microscopy and EQA for all laboratory personnel and all required reagents and equipment were supplied. On-site supervision was also conducted in accordance with the national EQA program three times a year, every 4 months by APHI [3]. Each round of rechecking and assessment was followed by feedback in the form of a written report, showing details of incorrect results and offering suggestions for quality improvement. This evaluation showed that EQA participated health facilities were achieved remarkable improvements to provide accurate and reliable malaria microscopic diagnosis service. The average percent agreement of detection and species identification, between participated health facility and APHI, were improved from 93.88 to 99.24% and 92.67 to 97.35% respectively.

This finding was in line with the report from Pakistan that showed external quality assessment and supervision approach was found to be feasible and acceptable to improve the quality of malaria microscopy service [7]. However, the overall agreement on detection of malaria parasite and identification of malaria species were higher than a study done in Hawassa, the southern part of Ethiopia, which showed 88 and 74.3% respectively. This variation could be due to assessment method difference that means our evaluation method was rechecking whereas their assessment method was proficiency testing [10].

Laboratories that reporting false positive results were lower in the last round of external quality assessment than in the previous three rounds. These findings were in agreement with the study conducted in the Democratic Republic of the Congo that the number of laboratories reporting false positive result was lower in the second assessment than the previous assessment [8]. This indicates that supportive site visits offered an opportunity to assess the actual condition and skills practiced in the laboratory and provide problem-solving strategies, corrective action and onsite training based on the identified gaps and the previous assessment feedback. This made the laboratories to maintain and improve the good performance of malaria microscopy diagnosis.

In the present study, the overall discordant result was 6.76% which was higher than reported from Pakistan 0.5 to 1% discordant slides. The difference may be due to the extent of external quality assessment implementation program and there may be also a difference in assessment method [7].

The current evaluation showed high false positive rate in four consecutive external quality assessment rounds from 10.5 to 0.79%. This finding was in line with the report from Hawassa [10], Canada [11], UAS [12] that showed 6.9, 2, and 7.8% respectively. However, it is lower than a finding from Democratic Republic of Congo [13] that showed 24.6%. The variation could be due to different assessment methods. These false positive results suggest that laboratory personnel often incorrectly report the presence of parasites; this could lead to unnecessary treatment or a delayed diagnosis of the true cause of illness and distract the clinician from considering other causes of fever and disease.

An accurate, correct laboratory diagnosis is essential as false negatives can result in untreated malaria patients and potentially severe consequences, including death. False negatives can also significantly undermine both clinical confidences in laboratory results and credibility in the community. False positive results are equally problematic. Patients presenting with fever not caused by malaria may be misdiagnosed and the true cause of their fever not treated. This can also have severe consequences, including the death of the patient. In addition, misdiagnosis of malaria will result in the unnecessary prescription of high-cost drugs and the unnecessary exposure of the patient to potentially toxic drugs. This is a needless burden to both the patient and the medical services [5].

#### Conclusion

The health facilities achieved remarkable improvements in quality service after participation in malaria external quality assessment program. The average percent agreement was 96.6% from the collected slides in four EQA rounds and the percent agreement became improved in each consecutive round and had a very good agreement (99.24%) with 0.79% false positive rate, 0.74% false negative rate and 97.35% agreement in species identification for *P. falciparum* at fourth round. Therefore, we recommend that to maintain and expand malaria external quality assessment program in the region to assure quality malaria microscopy service.

## Limitation

Due to inconsistent record management, the study could not evaluate the performance of health facilities regarding the quality of blood film preparation and staining procedures. It did not also assess associated factors for discordant results.

## Abbreviation

EQA: external quality assessment

## References

[1] World Health Organization. World Malaria Report 2016. Licence: CC BY-NC-SA 3.0 IGO. Geneva: World Health Organization.

[2] Federal Democratic Republic of Ethiopia Minister of Health. National malaria strategic plan 2014–2020. Addis Ababa: 2014.

[3] Ethiopian Health and Nutrition Research Institute Ethiopian Federal Ministry of Health. Manual for the laboratory diagnosis of malaria, First Edition. Ethiopian Health and Nutrition Research Institute Ethiopian Federal Ministry of Health; 2012.

[4] Fantahun B, Yeshambel B, Jemal A (2014). Does the practice of blood film microscopy for detection and quantification of malaria parasites in northwest Ethiopia fit the standard?. BMC Health Serv Res.

[5] World Health Organization. Malaria microscopy quality assurance manual. Version 1. World Health Organization; 2009.

[6] Ethiopian Health and Nutrition Research Institute Federal Ministry of Health. Malaria laboratory diagnosis external quality assessment scheme guideline; 2009.

[7] Muhammad AK, John DW, Muhammad A (2011). Munir District level external quality assurance (EQA) of malaria microscopy in Pakistan: pilot implementation and feasibility. Malaria J..

[8] Pierre M, Philippe G, Albert L (2013). External quality assessment of giemsa-stained blood film microscopy for the diagnosis of malaria and sleeping sickness in the Democratic Republic of the Congo. Bull World Health Organ.

[9] Amhara Regional Health Bureau. Regional malaria Annual Report. Bahir Dar: Ethiopia; 2013.

[10] Ayalew F, Tilahun B, Taye B (2014). Performance evaluation of laboratory professionals on malaria microscopy in Hawassa Town, Southern Ethiopia. BMC Research Notes..

[11] Thomson S, Lohmann RC, Crawford L (2000). External quality assessment in the examination of blood films for malarial parasites within Ontario, Canada. Arch Pathol Lab Med..

[12] Edson ED, Glick T, Massey L (2010). Detection and identification of malaria parasite: a review of proficiency test results and laboratory practice. Lab Med..

[13] Mukadi P, Gillet P, Lukuka A (2011). External quality assessment of malaria microscopy in the Democratic Republic of Congo. Malaria J..

